# Dendritic cells mediated by small extracellular vesicles derived from MSCs attenuated the ILC2 activity via PGE2 in patients with allergic rhinitis

**DOI:** 10.1186/s13287-023-03408-2

**Published:** 2023-07-24

**Authors:** Xiao-Qing Liu, Ya-Qi Peng, Long-Xin Huang, Chan-Gu Li, Peng-Peng Kuang, De-Hua Chen, Zi-Cong Wu, Bi-Xin He, Zhi-Rou Zhou, Qing-Ling Fu

**Affiliations:** 1 Otorhinolaryngology Hospital, The First Affiliated Hospital, Sun Yat-sen University, 58 Zhongshan Road II, Guangzhou, 510080 Guangdong People’s Republic of China; 2 Division of Allergy, The First Affiliated Hospital, Sun Yat-sen University, Guangzhou, People’s Republic of China; 3 Department of Otolaryngology-Head and Neck Surgery, Guangdong Provincial People’s Hospital (Guangdong Academy of Medical Sciences), Southern Medical University, Guangzhou, People’s Republic of China

**Keywords:** Small extracellular vesicles, Mesenchymal stromal cells, Dendritic cells, Group 2 innate lymphoid cells, Prostaglandin E2, Allergic rhinitis

## Abstract

**Background:**

Mesenchymal stromal cells-derived small extracellular vesicles (MSC-sEVs) have recently attracted considerable attention because of their therapeutic potential in various immune diseases. We previously reported that MSC-sEVs could exert immunomodulatory roles in allergic airway inflammation by regulating group 2 innate lymphoid cell (ILC2) and dendritic cell (DC) functions. Therefore, this study aimed to investigate the indirect effects of MSC-sEVs on ILC2s from patients with allergic rhinitis (AR) via DCs.

**Methods:**

Here, we isolated sEVs from induced pluripotent stem cells-MSCs using anion-exchange chromatography and mature DCs (mDCs) were treated with MSC-sEVs. sEV-mDCs were co-cultured with peripheral blood mononuclear cells from patients with AR or purified ILC2s. The levels of IL-13 and GATA3 in ILC2s were examined by flow cytometry. Bulk RNA sequence for mDCs and sEV-mDCs was employed to further probe the potential mechanisms, which were then validated in the co-culture systems.

**Results:**

sEV-mDCs showed impaired capacity in priming the levels of IL-13 and GATA3 in ILC2s when compared with mDCs. Furthermore, there was higher PGE2 and IL-10 production from sEV-mDCs, and the blockade of them especially the former one reversed the inhibitory effects of sEV-mDCs.

**Conclusions:**

We demonstrated that MSC-sEVs were able to dampen the activating effects of mDCs on ILC2s in patients with AR. Mechanismly, the PGE2-EP2/4 axis played an essential role in the immunomodulatory effects of sEV-mDCs on ILC2s. Herein, we provided new insights into the mechanism underlying the therapeutic effects of MSC-sEVs in allergic airway inflammation.

**Supplementary Information:**

The online version contains supplementary material available at 10.1186/s13287-023-03408-2.

## Introduction

Allergic rhinitis (AR) is a common disease globally with a prevalence of up to 25% in children and 40% in adults [1]. It has been generally believed that AR is a type 2 inflammation dominant allergic disease, mainly elicited by group 2 innate lymphoid cells (ILC2s), T helper 2 (Th2) cells, and dendritic cells (DCs), and epithelial cells [2–5]. ILC2s produced a profound amount of type 2 cytokines including IL-13 and IL-5, which were increasingly recognized as vital components in the pathogenesis of allergic diseases rather than Th2 cells. Moreover, we have previously reported that IL-33 producing myeloid DCs could promote the function and proliferation of ILC2s in patients with AR [6]. Together, these all indicated the important role of DC-ILC2 crosstalk in AR. Therefore, repressing the interplay between DCs and ILC2s might be a promising therapeutic strategy for AR.

Mesenchymal stromal or stem cells (MSCs) are multipotent stromal cells, which have shown broad and extensive immunomodulatory effects in various diseases [7]. Our group previously reported their therapeutic actions in allergic airway inflammation [8, 9]. MSC-derived small extracellular vesicles (MSC-sEVs) have received increased attention for their superior biosafety, lower immunogenicity and easier storage and transportation in recent years [10–12]. Moreover, MSC-sEVs exert extensive immunomodulatory capacity on multiple immune cells [13, 14], parallel or even enhanced than their parental cells. In addition, in order to facilitate clinical application in the future, anion-exchange chromatography reported in our previous study [15] can be a prospective strategy for mass or scalable production of MSC-sEVs.

It is well known that DCs, as the most powerful antigen presenting cells (APCs), play an important role in inducing allergic immune responses [16–18]. Our previous study confirmed that human myeloid DCs activated ILC2s in patients with AR through IL-33/ST2 axis [6], suggesting a new light on the roles of DCs and ILC2s in allergic pathogenesis. In view of this, the DC-ILC2 pathway may constitute a potent therapeutic target for AR. Beyond that, we have reported that MSC-sEVs could prevent ILC2 function via miR-146a-5p to alleviate allergic airway inflammation [15], which provided experimental evidence for MSC-sEVs as a novel cell-free therapeutic strategy in the allergic airway inflammation. Meanwhile, it has been reported in some studies that MSC-sEVs could affect the maturation and differentiation of DCs and induce tolerogenic DCs with impaired capacity of T cells stimulation [19–21]. Nevertheless, what is not yet clear is whether MSC-sEVs exert therapeutic effects on the activated DC-ILC2 interplay in allergic rhinitis. Based on the above, how DCs treated with MSC-sEVs regulate the function of ILC2s in patients with AR remains to be elucidated.

In this study, we investigated the effects of DCs treated with sEVs on ILC2s in patients with allergic rhinitis and further explored the underlying mechanisms. Our study aimed to provide new perspectives for the therapeutic potential of MSC-sEVs in the allergic airway inflammation.

## Methods

### Isolation and identification of MSC-sEVs

MSC-sEVs were isolated using anion-exchange chromatography as we previously reported [15]. In brief, human iPSC-MSCs were cultured in 150 cm^2^ cell culture plates and were replaced by chemically defined and protein-free (CDPF) medium for 48 h to generate the sEVs-enriched medium. Then, the medium was purified and concentrated by anion-exchange resin (Mylab Biotec, Guangzhou, Guangdong, China) and Pierce™ protein concentrator (30 kDa, Thermo Fisher Scientific, Rockford, IL, USA) to obtain MSC-sEVs, which were subsequently identified using transmission electron microscopy (TEM) (H7650, HITACHI, Tokyo, Japan), NanoSight NS300 (NanoSight Ltd, Navato, CA, USA), and western blot analysis for CD9, CD63 and Calnexin (all antibodies from Abcam, Cambridge, UK) detection. MSC-sEVs were stored at -80 °C for future studies.

### Subjects and cell isolation

Blood samples from patients with AR (*n* = 22) were obtained from The First Affiliated Hospital, Sun Yat-sen University. This study was approved by The Ethics Committee of The First Affiliated Hospital, Sun Yat-sen University, and informed consents were obtained from all participants. Human buffy coats of volunteers were from Guangzhou Blood Center, exemption of written informed consent was approved.

Human peripheral blood mononuclear cells (PBMCs) from patients with AR or buffy coats of volunteers were isolated using Ficoll (MP Biomedicals, Santa Ana, Calif) density gradient centrifugation. PBMCs from buffy coats were used for the generation of DCs and the sort of ILC2s. PBMCs from patients with AR were co-cultured with DCs and DCs treated with sEVs.

### Generation of DCs in vitro

DCs were generated from PBMCs in buffy coats according to our previous study [6]. In brief, CD14^+^ monocytes were isolated from PBMCs using CD14 MicroBeads (Miltenyi Biotec, Bergisch Gladbach, Germany), and cells were grown in RPMI 1640 medium containing 10% fetal bovine serum and 25 ng/mL GM-CSF (PeproTech, Inc, Rocky Hill, NJ), 5 ng/mL IL-4 (R&D Systems, Minneapolis, Minn) to differentiate into monocytes DCs (moDCs) in 5% CO2 at 37 °C for 5 days. Cells were then stimulated with 100 ng/mL LPS (Sigma-Aldrich, St Louis, Mo) or with LPS and 3 × 10^9^/mL MSC-sEVs for an additional 2 days to obtain mature (mDCs) or sEV-mDCs. In some experiments, sEV-mDCs were treated with MF63 (1 μM; MCE, Shanghai, China) on day 6 so-called MF63-sEV-mDCs. DCs were harvested on day 7 for flow cytometry, bulk RNA sequence, RT-qPCR and co-culture experiments. Supernatants were collected for assays of IL-10 and PGE2.

### Isolation and expansion of ILC2s

Lineage negative cells were isolated from buffy coats by Lineage Cell Depletion Kit (Miltenyi Biotec, Bergisch Gladbach, Germany); then, ILC2s were further sorted as Lin^−^CD127^+^CRTH2^+^ cells on a FACS Aria instrument (Beckman Coulter, Brea, Calif). Sorted ILC2s were plated in 96-well round-bottom plates with X-ray (30 Gy) irradiated allogeneic PBMCs in Yssel’s medium (homemade) supplemented with 100 U/mL IL-2 (PeproTech, Inc, Rocky Hill, NJ). After 7 to 10 days, ILC2s were transfered into 24-well plate (1 mL/well) and half of the medium was replaced every two days until the number of cells was enough to use. Purified ILC2s were used for co-culture experiments.

### MSC-sEV uptake by DCs

To determine MSC-sEV uptake by DCs, mCherry-labeled MSC-sEVs were prepared as our previous study in which the sEV uptake by human ILC2s peaked at 12 h [15]. In this study, DCs on day 5 were co-cultured with 3 × 10^9^/mL mCherry-labeled MSC-sEVs for 12 h, collected, and MFI of mCherry in DCs was analyzed using flow cytometry. In another experiment, DCs were placed on Poly-L-Lysine treated glass coverslips for 30 min, then counterstained using 4ʹ, 6-diamidino-2-phenylindole (DAPI, Invitrogen, Eugene, OR, USA), and the images were obtained with Nikon C2 confocal microscope (Nikon, Tokyo, Japan).

### Co-culture experiments of DCs with PBMCs or ILC2s

mDCs or sEV-mDCs were harvested and washed twice, and then co-cultured with PBMCs from patients with AR or purified ILC2s (1:10) for 3 days. In some experiments, MF63 (0.1 μM, 1 μM), ONO-AE3-208/PF-04418948 (1 μM) (MCE, Shanghai, China) or anti-IL-10 monoclonal antibody (2 μg/mL; R&D Systems, Minneapolis, MN, USA) was added into co-cultures. Supernatants were collected for the analysis of the cytokine levels using enzyme linked immunosorbent assay (ELISA), and the cells were analyzed for IL-13^+^ILC2s and GATA3^+^ILC2s by flow cytometry.

### Flow cytometry

DCs were assessed using the antibodies of anti-CD11c-PC5.5; anti-CD80-FITC; anti-HLA-DR-APC-Cy7 and anti-CD86-PE-Cy7. PBMCs or ILC2s were stained with Fixable Viability Dye-eF506 and the following specific mAbs: Lineage cocktail (CD2, CD3, CD14, CD16, CD19, CD56, CD235a, FceR1)-FITC (all from eBioscience, San Diego, Calif, USA); anti-CRTH2-PE (BD Pharmingen, USA) and anti-CD127-PE-Cy7 (eBioscience, San Diego, Calif, USA). For intracellular cytokine assay, PBMCs or ILC2s were stimulated with phorbol myristate acetate (PMA), ionomycin, and brefeldin A (BFA) for 5 h, and then, the cells were fixed, permeabilized, and stained with anti-IL-13-BV421 (BioLegend, San Diego, CA, USA). For GATA3 levels detection, the cells were processed with the FoxP3/Transcription Factor Staining Buffer Set before staining with anti-GATA3-BV421(BioLegend, San Diego, CA, USA). After staining, samples were analyzed on a CytoFLEX Flow Cytometer (Beckman Coulter, Hercules, CA, USA).

### ELISA

The cell supernatants were analyzed by IL-13 and IL-10 ELISA kits (Invitrogen, Waltham, MA, USA) and PGE2 Assay Kit (R&D Systems, Minneapolis, MN, USA) according to the manufacturer’s instructions.

### RT-quantitative PCR

Total RNA isolated from DCs with RNAiso Plus reagent, and 5X PrimeScript RT Master Mix kit (all from TaKaRa, Kusatsu, Shiga, Japan) was used for converting 1 μg of total RNA to first-strand cDNA following the manufacturer’s instructions. The quantitative PCR of PTGES (sense primers, 5′-CTGGTCATCAAGATGTACGTGG-3′, and reverse primer, 5′-TTAGGACCCAGAAAGGAGTAGA-3′), IL-10 (sense primers, 5′-GTTGTTAAAGGAGTCCTTGCTG-3′, and reverse primer, 5′-TTCACAGGGAAGAAATCGATGA-3′) and TNFSF15 (sense primers, 5′-AGTTCCAGGCTCTAAAAGGAC-3′, and reverse primer, 5′-GCTTATCTCCGTCTGCTCTAAG-3′) were performed using the FastStart Universal SYBR Green Master kit (Roche, Mannheim, Germany). β-Actin (sense primers, 5′- AGAGCTACGAGCTGCCTGAC-3′, and reverse primer, 5′- AGCACTGTGTTGGCGTACAG-3′) was used as an endogenous reference. The PCR was performed as 10-min initial denaturation at 95 °C, with 40 cycles consisting of 10 s at 95 °C and 30 s at 60 °C carried out on the CFX96 Real-Time PCR cycler (Bio-Rad, Hercules, Calif). Expression of target gene was expressed as fold increase relative to the expression of β-actin. The mean value of the replicates for each sample was calculated and expressed as cycle threshold (Ct). The amount of gene expression was then calculated as the difference (ΔCt) between the Ct value of the target gene and the Ct value of β-actin. The relative target mRNA levels were determined as 2^−ΔCt^.

### Bulk RNA sequence

Total RNA was isolated from mDCs and sEV-mDCs using the Magzol Reagent (Magen, China) according to the manufacturer’s protocol, The quantity and integrity of RNA yield was assessed by using the K5500 (Beijing Kaiao, China) and the Agilent 2200 TapeStation (Agilent Technologies, USA) separately. Briefly, the mRNA was enriched by oligodT according to instructions of NEBNext® Poly(A) mRNA Magnetic Isolation Module (NEB, USA). And then fragmented to approximately 200 bp. Subsequently, the RNA fragments were subjected to first-strand and second-strand cDNA synthesis following by adaptor ligation and enrichment with a low-cycle according to instructions of NEBNext® Ultra™ RNA Library Prep Kit for Illumina. The purified library products were evaluated using the Agilent 2200 TapeStation and Qubit (Thermo Fisher Scientific, USA). The libraries were sequenced by Illumina (Illumina, USA) with paired-end 150 bp at Ribobio Co. Ltd (Ribobio, China).

### Statistical analyses

Differences between two groups were analyzed by the paired t test for the data with normal distribution, and the Wilcoxon matched-pairs signed-rank test was performed to compare the data with abnormal distribution. Three or more groups were compared using one-way repeated-measures ANOVA. *P < 0.05 was considered to be statistically significant. Analyses were performed using GraphPad Prism 8.0 software (GraphPad Software, La Jolla, Calif).

## Results

### Identification of MSC-sEV

**Fig. 1:**
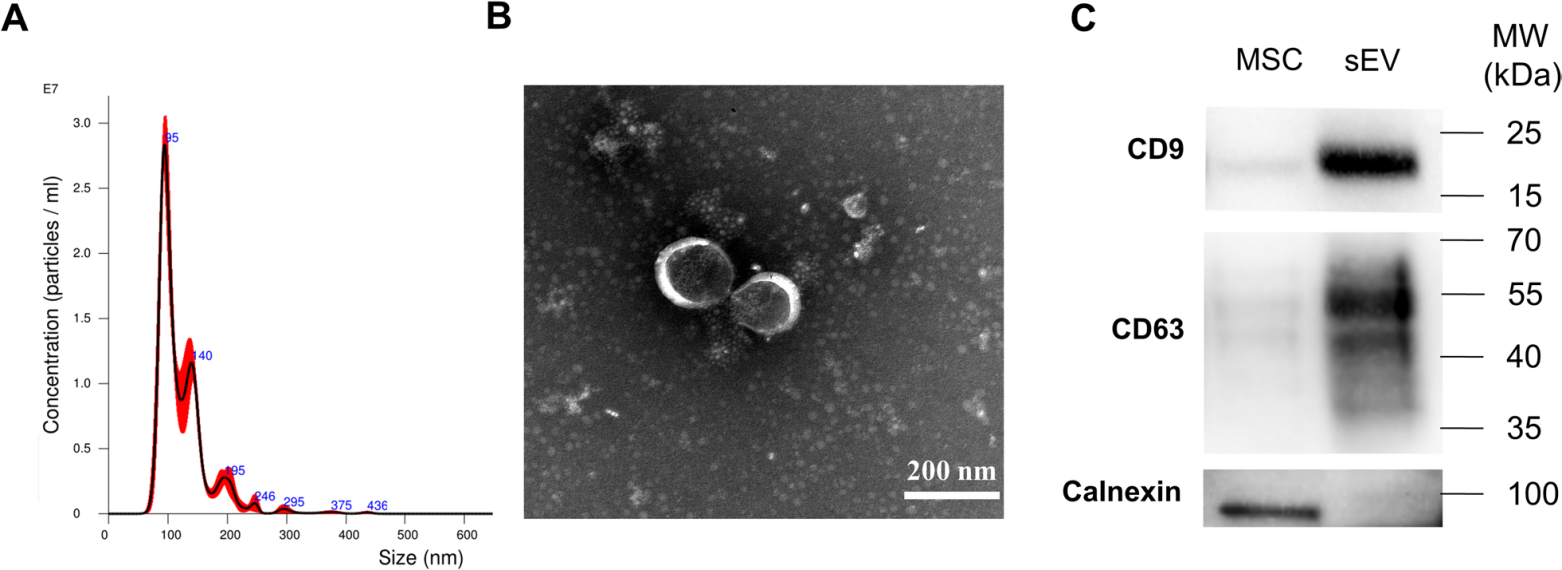
Identification of MSC-sEV. MSC-sEVs were isolated from iPSC-MSCs using anion-exchange chromatography. **A**. Nanoparticle Tracking Analysis (NTA) showed the particle size distribution and concentration of MSC-sEVs. **B**. TEM showed morphology and size of MSC-sEVs. **C**. CD9, CD63 and calnexin expression in MSCs and sEVs were determined by western blotting. The original blots are presented in Additional file 1: Fig. S4. MSC: mesenchymal stromal cells; MW: molecular weight; sEV: small extracellular vesicles; TEM: transmission electron microscope. Scale bar, 200 nm

MSC-sEVs were isolated from iPSC-MSCs using anion-exchange chromatography as we previously reported [15]. First, the Nanoparticle Tracking Analysis (NTA) showed MSC-sEVs mainly distributed with a size of approximately 100 nm (Fig. 1A), and TEM showed they were round in shape and had a lipid bilayer membrane structure (Fig. 1B). Moreover, CD9 and CD63 expression in MSC-sEVs was higher than their parent MSCs as assessed by western blotting. And MSC-sEVs did not express the endoplasmic reticulum protein calnexin which was positive in MSCs (Fig. 1C). Hence, MSC-sEVs in our study conformed to classical characteristics of exosomes.

### MSC-sEVs inhibited the effects of dendritic cells on ILC2s in patients with allergic rhinitis

**Fig. 2:**
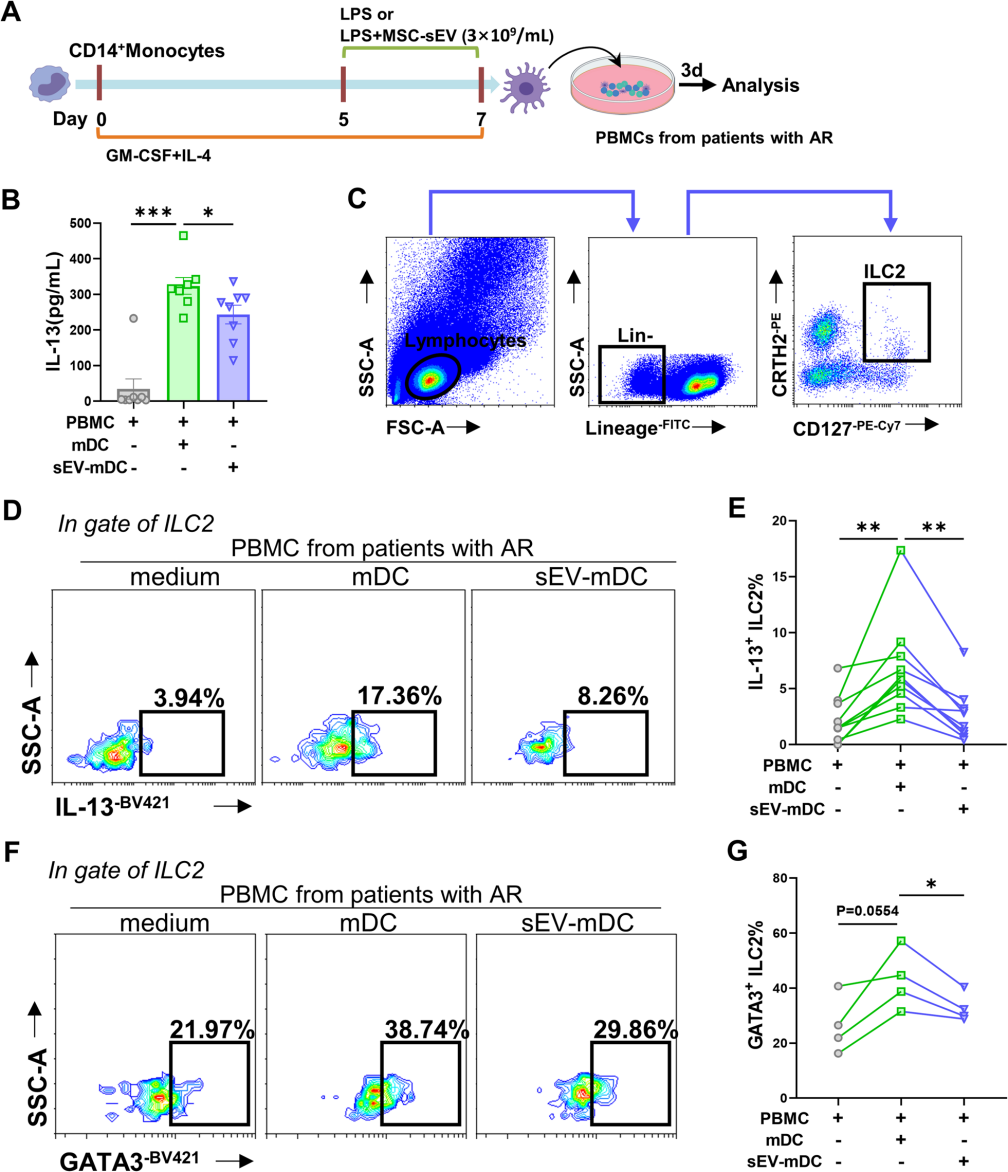
MSC-sEVs inhibited the effects of dendritic cells on ILC2s in patients with allergic rhinitis. **A**. Schematic representation of mDCs and sEV-mDCs generation and co-cultures. **B**–**F**. PBMCs from patients with AR were co-cultured with allogeneic mDCs or sEV-mDCs for 3 days. **B**. The levels of IL-13 in the supernatants were analyzed by ELISA (*n* = 8). **C**. Gating strategy of human ILC2s with Lin^−^CRTH2^+^CD127^+^. D-E. Intracellular IL-13 levels in ILC2s were analyzed by flow cytometry (*n* = 10). F-G. Levels of GATA3 in ILC2s were analyzed by flow cytometry (*n* = 4). DC: dendritic cell; ILC2: group 2 innate lymphoid cell; MSC: mesenchymal stromal cell; sEV: small extracellular vesicle. Data are shown as mean ± SEM. *P < 0.05, **P < 0.01, ***P < 0.001

mDCs were generated from CD14^+^monocytes in vitro as we previously reported [6], treated with sEVs as Fig. 2A, and were subsequently identified using flow cytometry. We found sEVs treatment did not affect DC phenotype and maturation status (Additional file 1: Fig. S1). Next, PBMCs were isolated from patients with AR and co-cultured with mDCs or sEV-mDCs, and co-culture supernatants were assayed after 3 days. IL-13 was elevated when co-cultured with mDCs and significantly decreased in the sEV-mDCs co-cultures (*P* < 0.05, Fig. 2B). ILC2s have been considered to be one of the major sources of IL-13 [22]. We further evaluated the effects of mDCs or sEV-mDCs on ILC2 function using flow cytometry. Human blood ILC2s were defined as Lin^−^CD127^+^CRTH2^+^ as done in our previous studies [6, 23] (Fig. 2C). We found that the levels of IL-13^+^ILC2s were increased after co-cultured with mDCs, which were consistent with our previous study [6]. However, there were lower levels of IL-13^+^ILC2s after the administration with sEV-mDCs compared with mDCs (*P* < 0.001, Fig. 2D, E). These findings suggested that sEV-mDCs showed impaired capacity in priming ILC2s; namely, MSC-sEVs were able to inhibit the facilitation of mDCs on ILC2s in patients with AR in vitro.

GATA3 is the key transcription factor of ILC2s and regulates the functional effector production in ILC2s [24, 25]. We next investigated the effects of mDCs or sEV-mDCs on GATA3 expression in ILC2s. Similarly, GATA3^+^ILC2s were significantly increased in response to mDCs. However, lower levels of GATA3^+^ILC2s were observed with the treatment with sEV-mDCs (*P* < 0.05, Fig. 2F, G). This provided further evidence for less activity of sEV-mDCs to activate ILC2s compared with mDCs.

### sEV-mDCs exhibited impaired capacity to prime ILC2 function

**Fig. 3:**
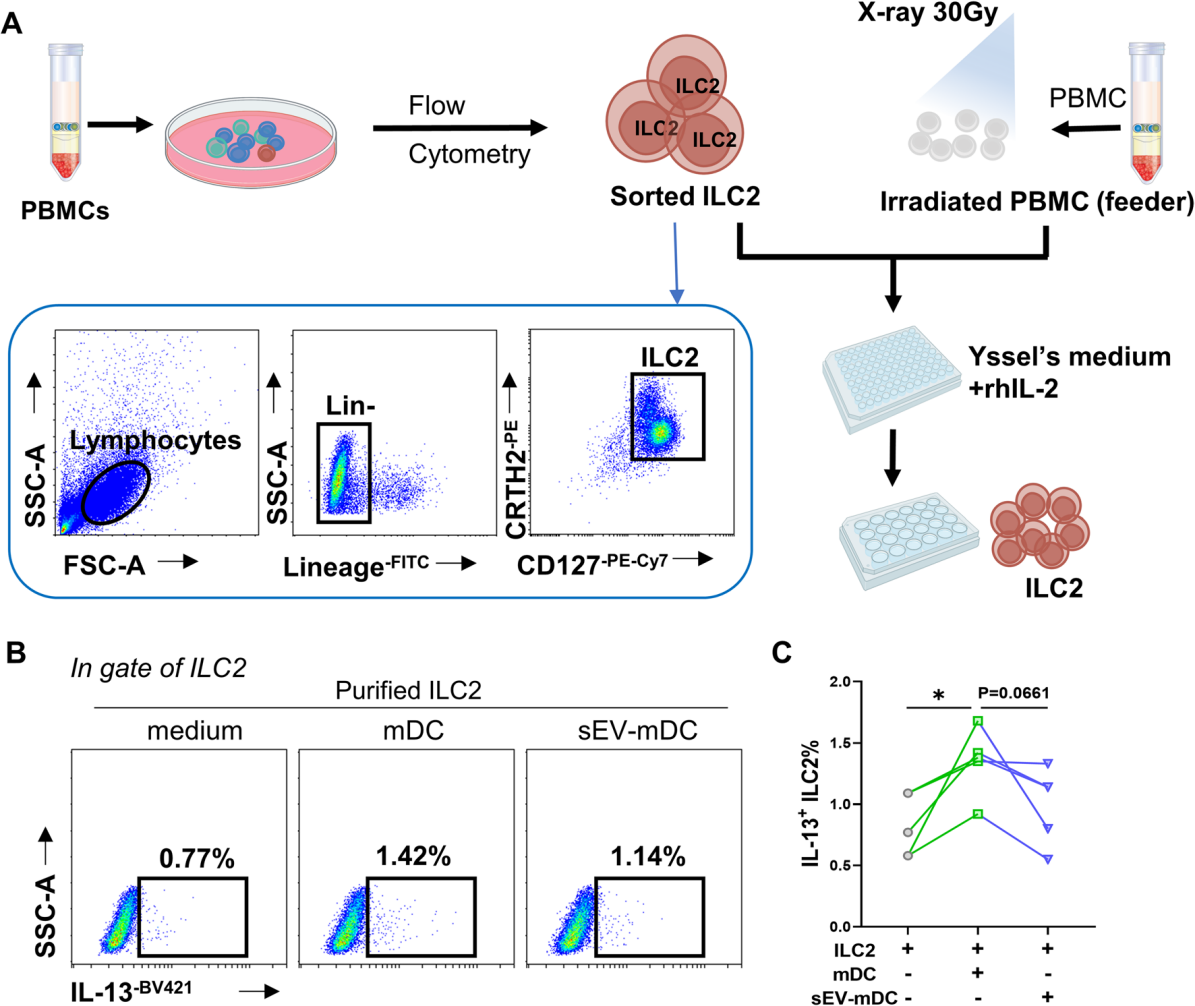
sEV-mDCs exhibited impaired capacity on purified ILC2 function. **A**. Schematic of isolation and amplifying purified ILC2s. **B**–**C**. Purified ILC2s were co-cultured with allogeneic mDCs or sEV-mDCs for 3 days. Intracellular IL-13 levels in ILC2s were analyzed by flow cytometry (*n* = 5). *P < 0.05

To further verify our findings above, we sorted purified ILC2s from the buffy coats of healthy human volunteers. Allogeneic PBMCs were irradiated with X-ray as feeders and co-cultured with flow sorted ILC2s, and 3–4 weeks later, expanded ILC2s were obtained for co-culture experiments (Fig. 3A). After co-culturing purified ILC2s with mDCs or sEV-mDCs, significantly increased IL-13^+^ILC2s in the mDCs co-cultures were detected, which were down-regulated by sEV-mDCs compared with mDCs but without significant difference (*P* = 0.0661, Fig. 3B, C).

### Uptake of MSC-sEV by DCs

**Fig. 4:**
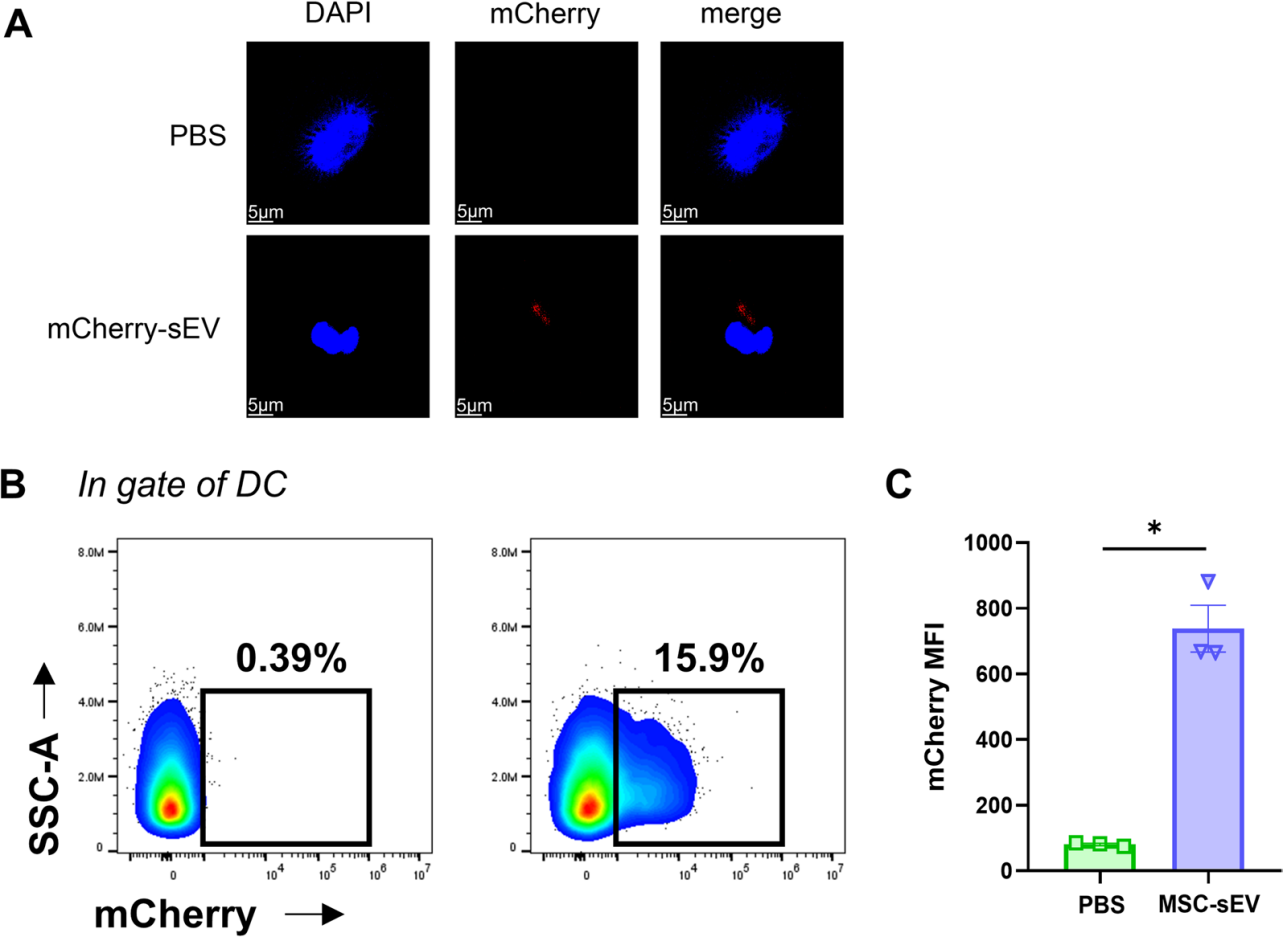
Uptake of MSC-sEV by DCs. DCs on day 5 were co-cultured with mCherry-labeled MSC-sEVs for 12 h. **A**. The cells were photographed by confocal microscopy. Panels showed mCherry (Red) and DAPI (Blue). **B**–**C**. mCherry in DCs was analyzed using flow cytometry. MFI: mean fluorescence intensity. Scale bar, 5 μm. Data are shown as mean ± SEM. *P < 0.05

The findings above have shown that MSC-sEVs dampened the activating effects of mDCs on ILC2s. We then investigated the cellular uptake of sEV by DCs using mCherry-labeled MSC-sEVs. We found that the uptake of sEV by DCs was significantly visualized using fluorescence microscopy (Fig. 4A) and further confirmed by flow cytometry (Fig. 4B–C). These results indicated that DCs were able to take up MSC-sEV directly.

### The different expression of PGE2 and IL-10 between sEV-mDCs and mDCs

**Fig. 5:**
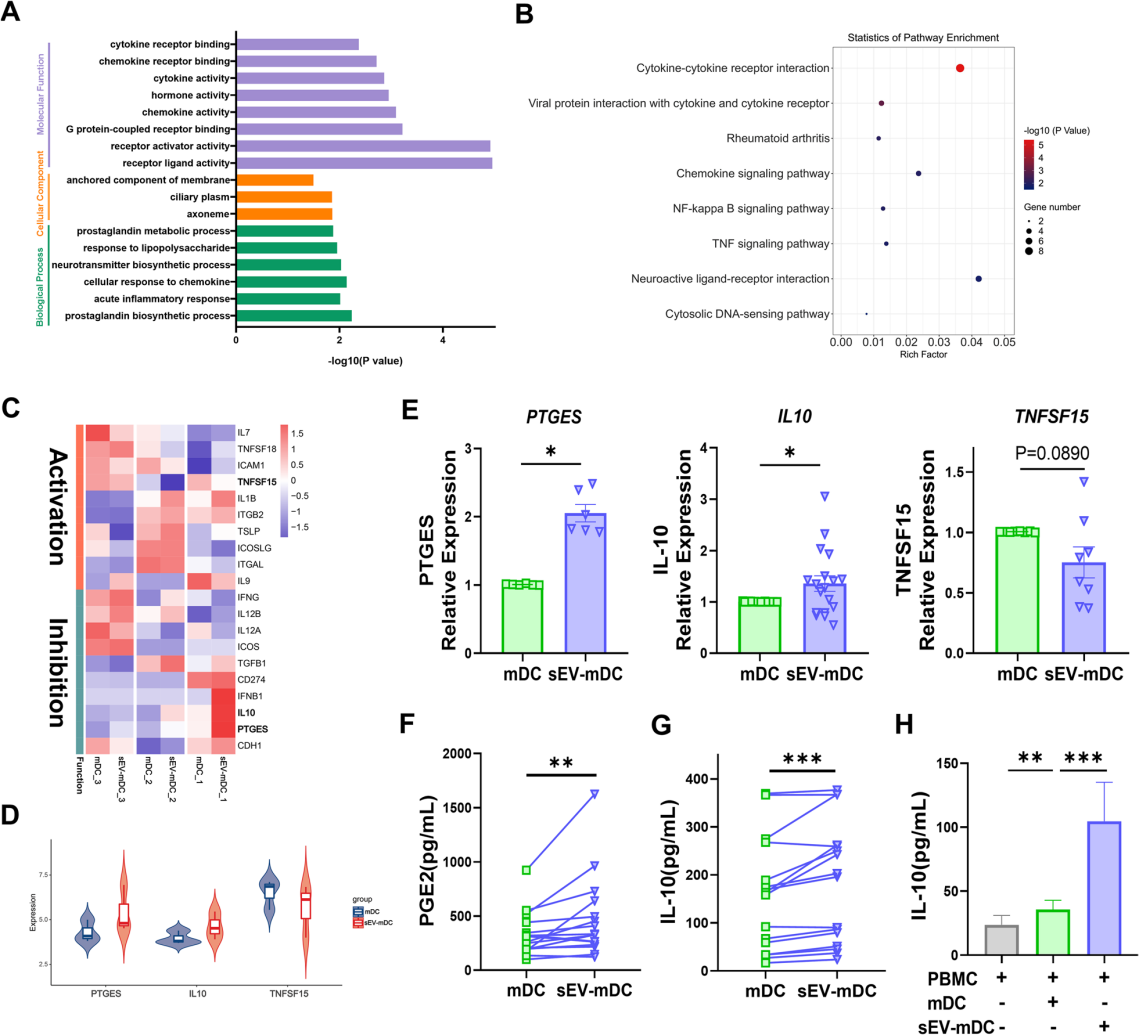
The expression of PGE2 and IL-10 in sEV-mDCs and mDCs. **A**–**B**. GO analysis and KEGG enrichment analysis of differential mRNAs between mDCs and sEV-mDCs. **C**. Heatmap representation of differential mRNAs involved in ILC2s regulation between mDCs and sEV-mDCs. **D**. The violin plot showed the differential expression of PTGES, IL10 and TNFSF15 in mDCs and sEV-mDCs. **E**. The levels of PTGES, IL10 and TNFSF15 were examined by RT-quantitative PCR (*n* = 6–18). F. The levels of PGE2 in the supernatants of mDCs and sEV-mDCs (*n* = 16). G-H. The levels of IL-10 in the supernatants of mDCs and sEV-mDCs and co-cultured PBMCs (*n* = 13–16). PTGES: Prostaglandin E Synthase; IL-10: Interleukin-10; PGE2: Prostaglandin E2. Data are shown as mean ± SEM. *P < 0.05, **P < 0.01, ***P < 0.001

In order to further investigate possible mechanisms underlying the functional differences on ILC2s between mDCs and sEV-mDCs, we performed bulk RNA sequence for mDCs and sEV-mDCs. In total, we identified 161 differentially expressed mRNAs between mDCs and sEV-mDCs. GO enrichment analysis revealed that those differentially expressed mRNAs were related to processes typically associated with inflammation, such as cytokine and its receptor signaling, receptor ligand activity and prostaglandin process. The KEGG pathway analysis showed similarly involving cytokine-cytokine receptor interaction (Fig. 5A, B), suggesting that MSC-sEVs influenced the cytokine production and interaction of mDCs, which might mediate the immunomodulatory effects of sEV-mDCs. We primarily focused 20 of mRNAs involved in regulating ILC2s function (Fig. 5C). Of these, PTGES, Prostaglandin E Synthase to catalyze PGE2 synthesis, and IL-10 were up-regulated in sEV-mDCs and they have already been shown to play an inhibitory role in ILC2s function [26–28]. In contrast, TNFSF15 is associated with ILC2s activation [29, 30] and was higher in mDCs (Fig. 5D). The different expression of mRNAs above were further validated with more samples using RT-qPCR. sEV-mDCs did show significantly higher expression of PTGES and IL-10 (*P* < 0.05, Fig. 5E). While TNFSF15 was down-regulated in sEV-mDCs but showed no significance (*P* = 0.089, Fig. 5E). However, there were no significant differences in the level of PD-L1, IFNG, TGFB, IL-6, ICOS-L and IL-7 mRNA between mDCs and sEV-mDCs (Additional file 1: Fig. S2). Furthermore, we performed validation for PGE2 and IL-10 at the protein level using ELISA. We found that sEV-mDCs secreted more PGE2 and IL-10 than mDCs significantly (Fig. 5F, G). In parallel, we observed a significantly increased level of IL-10 in the supernatants of co-cultures and it is even more obvious with the sEV-mDCs (*P* < 0.001, Fig. 5H). Taken together, these results suggested that increased production of PGE2 and IL-10 may be reasonable for the immunomodulatory effects of sEV-mDCs.

### PGE2 mediated the inhibitory function of sEV-mDCs through EP2/4 on ILC2s

To further determine whether PGE2 and IL-10 were involved in the immunomodulatory effects of sEV-mDCs on ILC2s, we attempted to block their interaction pathways during DC induction and in co-culture systems via decreasing PGE2 production and blocking the interaction between PGE2 with their receptors or anti-IL-10 antibody.

**Fig. 6:**
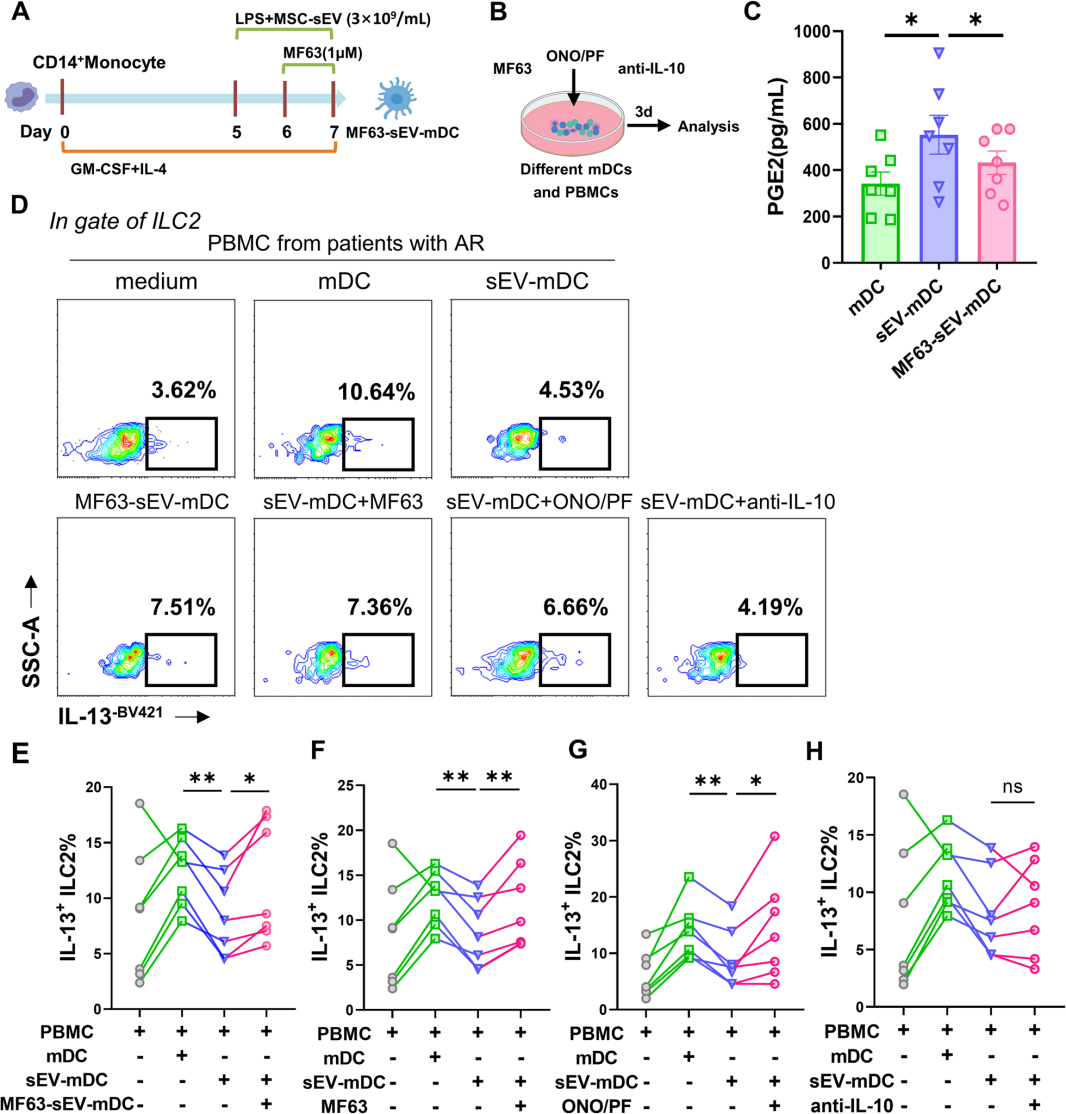
PGE2 mediated the impaired function of sEV-mDCs on ILC2s through EP2/4. PBMCs from patients with AR were co-cultured with allogeneic mDCs, sEV-mDCs, MF63-pretreated sEV-mDCs or combined with MF63 (0.1 μM), ONO/PF (1 μM), anti-IL-10 (2 μg/mL), respectively, for 3 days. **A–B**. Schematic of MF63-sEV-mDCs induction and blocking experiments. **C**. The levels of PGE2 in the supernatants of mDCs, sEV-mDCs and MF63-sEV-mDCs (*n* = 7). **D**. Intracellular IL-13 levels in ILC2s under different condition were analyzed by flow cytometry. **E–H**. Percentage of IL-13^+^ILC2s in the culture conditions as described (*n* = 7). MF63: pharmacological inhibitor of prostaglandin E Synthase; ONO/PF: ONO-AE3-208 and PF-04418948, antagonists for EP2/EP4. Data are shown as mean ± SEM. *P < 0.05, **P < 0.01

PGE2 synthase (PTGES) has been known as an inducible enzyme, which could convert PGH to PGE2, the terminal product. In our study, MF63, a potent selective inhibitor of human PTGES, was used to block or inhibit the production of PGE2 in sEV-mDCs. Firstly, we pretreated sEV-mDCs with MF63 to generate MF63-sEV-mDCs, washed and then co-cultured them with PBMCs (Fig. 6A). The levels of PGE2 in the supernatants of MF63-sEV-mDCs, as expected, were suppressed obviously (*P* < 0.05, Fig. 6C). After co-cultured with PBMCs from AR patients, MF63-sEV-mDCs led to a marked increase in IL-13^+^ILC2s (*P* < 0.05, Fig. 6D, E). In order to further confirm the above results, PBMCs were co-cultured with sEV-mDCs with the administration of MF63 in the co-culture system (Fig. 6B). Similarly, we found that there were higher levels of IL-13^+^ILC2s in the co-culture of MF63 with two different concentration of 0.1 μM (*P* < 0.01, Fig. 6D, F) and 1 μM (Additional file 1: Fig. S3A) and sEV-mDCs compared to sEV-mDCs alone (*P* < 0.01, Fig. 6D, F). These findings have shown that MF63 reversed the impaired effects of sEV-mDCs, indicating the key role of PGE2 for the low stimulating effects of sEV-mDCs on ILC2s.

PGE2 was reported to suppress ILC2 function through the engagement of both EP2 and EP4 receptors [26]. We used selective EP2 (PF-04418948) and EP4 (ONO-AE3-208) receptor antagonists to block the binding of PGE2 with its receptors of EP2 and EP4 on ILC2s. ONO-AE3-208/PF-04418948 were added into co-cultures of PBMCs and sEV-mDCs (Fig. 6B). We found that there were higher levels of IL-13^+^ILC2s under the administration of ONO-AE3-208/PF-04418948 compared to sEV-mDCs (*P* < 0.05, Fig. 6D, G). It further indicates the role of PGE2 in the effects of sEV-mDCs on ILC2 function.

We next used anti-IL-10 antibody to check the role of IL-10 in the low activity of sEV-mDCs on ILC2 stimulation. However, we did not observe any effects of anti-IL-10 antibody on the level of IL-13^+^ILC2s compared to sEV-mDCs alone (Fig. 6H). Nevertheless, we found that anti-IL-10 antibody reversed IL-13 production in the mixed co-cultures in which IL-13 production may be derived from several kinds of cells but not only ILC2s (Additional file 1: Fig. S3B). Totally, these results indicated that sEV-mDCs exerted immunosuppressive effects on ILC2s via increased PGE2 acting on EP2/4 receptors.

## Discussion

**Fig. 7:**
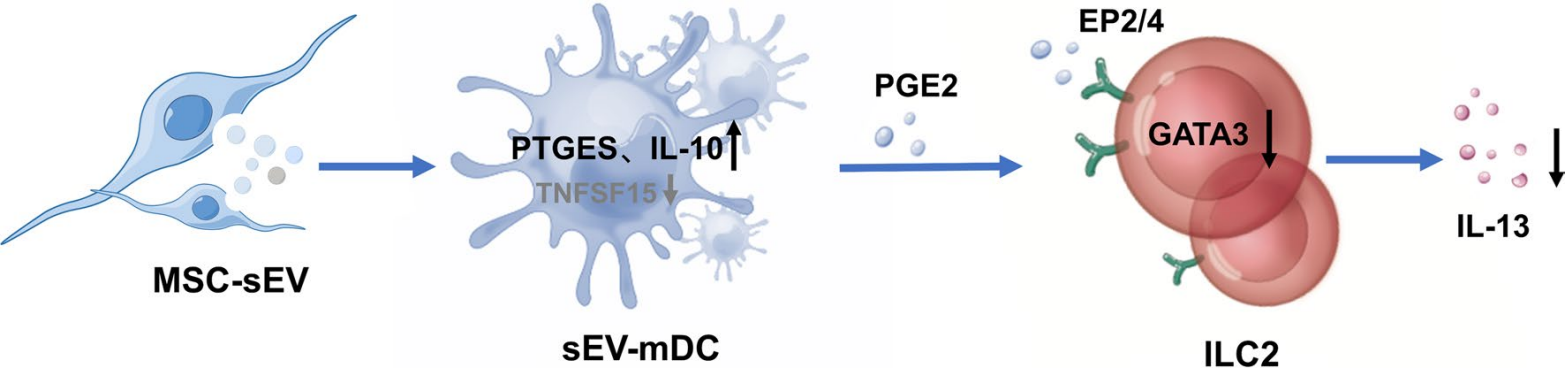
Schema for MSC-sEVs indirectly suppress ILC2 function in allergic rhinitis through increasing production of PGE2 from sEV-mDCs

In this study, we generated sEV-mDCs from mDCs with MSC-sEVs treatment and investigated the effects of sEV-mDCs on ILC2s in patients with allergic rhinitis. We demonstrated that MSC-sEVs were able to dampen the activating effects of mDCs on ILC2s in patients with AR, and the immunomodulatory mechanisms involved were mainly mediated by PGE2 (Fig. 7). Our findings provide new insights into the mechanism underlying the therapeutic effects of MSC-sEVs in allergic airway inflammation and further support the promising prospect for MSC-sEVs to be a novel cell-free therapeutic strategy in the allergic disease.

Recently, there has been growing attention in MSC-derived extracellular vesicles for their roles in many diseases. Prior studies have noted the therapeutic effects of MSC-sEVs in allergic airway inflammation via several mechanisms, such as immunoregulation on pulmonary macrophages [31], releasing miR-146a-5p to suppress the function of ILC2s [15] and promoting the proliferation and immunosuppression capacity of regulatory T cells, which may be mediated by APCs [32]. As the innate phenocopy of Th2 cells, ILC2s play a critical role in the pathogenesis of allergic disease and they can be activated by DCs [6, 33]. Given that the DC-T cells interaction pathway provides significant contribution to allergic disease [34] and MSC-sEVs may regulate T cell responses by affecting DCs [35]. Hence, it could conceivably be hypothesized that MSC-sEVs are able to regulate ILC2s function through DCs in allergic airway inflammation.

To figure out the effects of MSC-sEVs on the DC-ILC2 pathway, human mature DCs treated with sEV were co-cultured with PBMCs from AR patients. We identified that sEV-mDCs had weak activity to stimulate ILC2 function with the production of IL-13 in ILC2s. Simultaneously, there was a decreased level of GATA3 in ILC2s after co-culturing with sEV-mDCs. Further, we examined the findings in the purified ILC2s co-cultures with sEV-mDCs and mDCs, and obtained consistent results as well. Taken together, these findings elucidated that MSC-sEVs could inhibit the function of ILC2s by decreasing the activating function of mDCs. However, we found that sEVs treatment did not affect DC mature phenotypes. Regarding the effects of MSC-sEVs on DC maturation, the results varied among different studies. When accessing the expression of CD80, CD86 and HLA-DR et al., MSC-sEVs were reported to show inhibitory effects or no significantly effects [20, 21]. Such phenotypic differences might be due to the different sources of DCs (mice or human) or different doses of MSC-sEVs and experimental conditions. In this study, mDCs mediated by MSC-sEVs showed no significant differences in the maturation phenotypes, but they displayed impaired capacity to prime ILC2 function in allergic rhinitis. These results were consistent with our previous study that MSCs did not affect the phenotype of mature DCs but modulated their functional properties by increasing their phagocytic ability [36].

Mechanistically, we performed bulk RNA sequence to evaluate the different mRNAs related to ILC2s regulation between sEV-mDCs and mDCs. We observed increased level of PTGES and IL-10 and lower TNFSF15 expression in sEV-mDCs, and the former two were validated in RT-qPCR analyses. PTGES is one of PGE synthases to catalyze PGE2 synthesis, and it shows inducible expression in immune cells and is up-regulated in inflammation and tumor environment [37]. It is reported that PTGES/PGE2 signaling promotes immunosuppression in tumor microenvironment [38]. PGE2 and IL-10 are two of the main effectors of MSC-mediated immunosuppression [39]. Moreover, we previously determined the immunomodulatory properties of iPSC-MSCs and BM-MSCs were associated with PGE2 in allergic rhinitis [40]. In addition, we and others have previously reported MSCs and their sEVs were capable to induce IL-10 production in DCs [21, 36]. As expected, the elevated protein level of PGE2 and IL-10 in the supernatants from sEV-mDCs was determined using ELISA. Thus, it is reasonable to speculate that PGE2 and IL-10 may mediate the immunomodulatory effects of sEV-mDCs.

To confirm the role of PTGES/PGE2 and IL-10 in the functional sEV-mDCs, some antagonists or antibodies were employed. The MF63-sEV-mDCs with decreased secretion of PGE2 did not exhibit suppressive effects on IL-13^+^ILC2s. Similarly, the inhibitory effects of sEV-mDCs on ILC2s were reversed by adding MF63 or PGE2 receptor antagonists into the co-cultures. Overall, we proved that PGE2 played a critical role in the immunomodulatory effects of sEV-mDCs on ILC2s. Though the application of anti-IL-10 antibody suggested that IL-10 might not contribute to the inhibitory function of sEV-mDCs on ILC2s, increased IL-13 level in the co-culture supernatants indicated IL-10 might involve in suppression of sEV-mDCs on other IL-13-producing cells than ILC2s, which should be study in future research.

Besides DCs, increased IL-10 secretion in the co-cultures might also originate from other sources such as Tregs. On the basis of our previous studies that MSCs regulate type 2 innate lymphoid cells via regulatory T cells through ICOS-ICOSL interaction and IL-10 production [41], it can be presumed that sEV-mDCs may induce more IL-10 producing regulatory T cells which can suppress ILC2s synergistically. We will need to elucidate this possibility in the future experiments.

We acknowledge that there are some limitations in this study. We showed that MSC-sEVs were able to dampen the activation of dendritic cells on ILC2s through PGE2 in vitro experiments. However, in vivo evidence to support this conclusion is lacking at the moment. Further studies are needed to confirm and validate these findings by transferring mDCs and sEV-mDCs in ILC2-dominant allergic airway inflammation mouse models.

## Conclusion

Taken together, the findings of our study suggested that MSC-sEVs were able to inhibit the effects of mDCs on ILC2s in allergic rhinitis, and PGE2 played a critical role in it. These results have significant implications for more comprehensive understanding of therapeutic mechanisms of MSC-sEVs in allergic diseases.

## Supplementary Information

**Additional file 1.** Supplementary Figures.

## Data Availability

The data that support the findings of this study are available from the corresponding author upon reasonable request. The RNA sequence data have been deposited at the NCBI Gene Expression Omnibus (GEO; https://www.ncbi.nlm.nih.gov/geo/) under accession number GSE229003.

## Abbreviations

AR: Allergic rhinitis
APCs: Antigen presenting cells
DCs: Dendritic cells
ILC2s: Group 2 innate lymphoid cells
IL-10: Interleukin-10
MSCs: Mesenchymal stromal cells
NTA: Nanoparticle tracking analysis
PBMCs: Peripheral blood mononuclear cells
PGE2: Prostaglandin E2
PTGES: Prostaglandin E synthase
sEVs: Small extracellular vesicles
TEM: Transmission electron microscope
Th2: T helper 2 cell
