# Supplementary material for: Dendritic cells mediated by small extracellular vesicles derived from MSCs attenuated the ILC2 activity via PGE2 in patients with allergic rhinitis
Source: Stem Cell Res Ther. 2023 Jul 24;14:180. doi: 10.1186/s13287-023-03408-2 (PMC10367306; doi:10.1186/s13287-023-03408-2)
Supplement: Supplementary file 1 — Additional file 1. Supplementary Figures. [file 13287_2023_3408_MOESM1_ESM.docx]

Dendritic cells mediated by small extracellular vesicles derived from MSCs attenuated the ILC2 activity via PGE2 in patients with allergic rhinitis

Xiao-Qing Liu^1,2†^, Ya-Qi Peng^3†^, Long-Xin Huang^1,2^, Chan-Gu Li^1,2^, Peng-Peng Kuang^1,2^, De-Hua Chen^1,2^, Zi-Cong Wu^1^, Bi-Xin He^1,2^, Zhi-Rou Zhou^1,2^, Qing-Ling Fu^1,2*^

^1^ Otorhinolaryngology Hospital, The First Affiliated Hospital, Sun Yat-sen University, Guangzhou, China

^2^ Division of Allergy, The First Affiliated Hospital, Sun Yat-sen University, Guangzhou, China

^3^ Department of Otolaryngology-Head and Neck Surgery, Guangdong Provincial People's Hospital (Guangdong Academy of Medical Sciences), Southern Medical University, Guangzhou, China

^†^ These authors have contributed equally to this work and share first authorship.

^*^**Correspondence:**Prof. Qing-Ling Fu

fuqingl@mail.sysu.edu.cn

**SUPPLEMENTARY FIGURES**

**
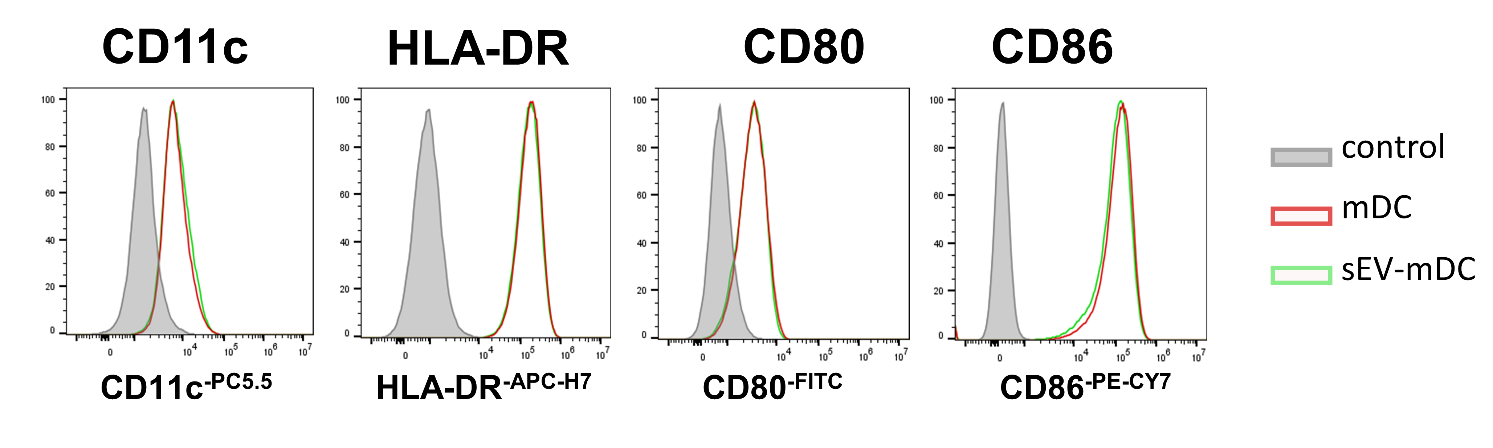
Supplementary Fig. 1 sEVs treatment did not affect DC mature phenotypes.** Expression of surface markers CD11c, HLA-DR, CD80 and CD86 on mDCs and sEV-mDCs as determined by flow cytometry.


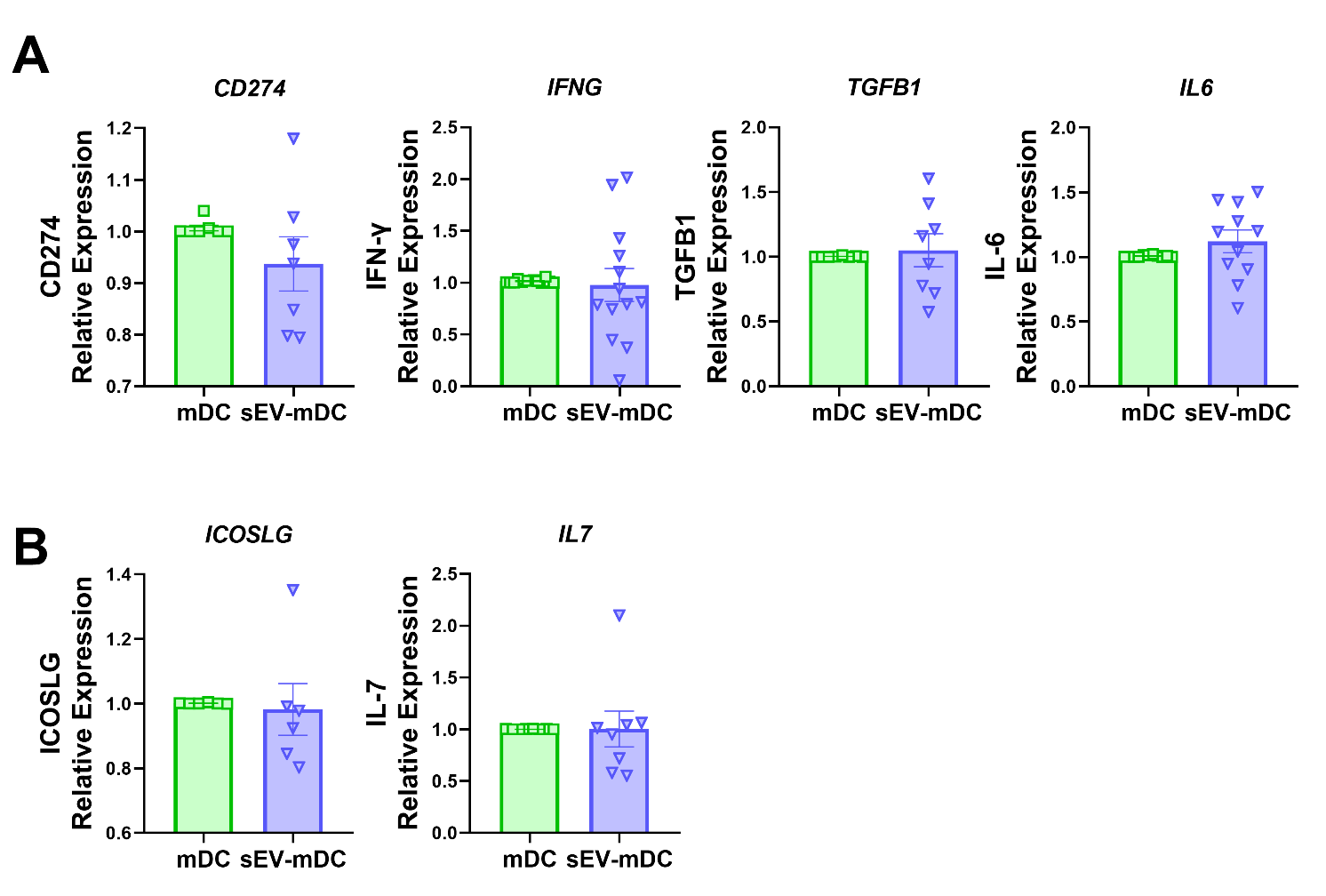


**Supplementary Fig. 2 The mRNA levels of some genes related to ILC2 function in DCs.**  A. ILC2 inhibition-related genes CD274, IFNG, TGFB1 and IL6, B. ILC2 activation-related genes ICOSLG and IL7 expression in mDCs and sEV-mDCs examined by RT-qPCR.


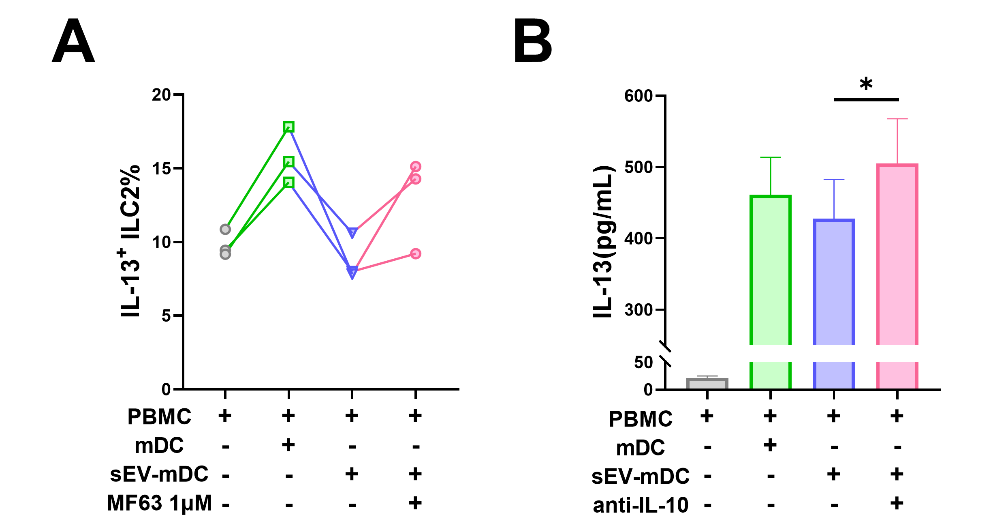


**Supplementary Fig. 3 The role of MF63 or anti-IL-10 antibody in the effects of sEV-mDCs on ILC2 function.** A. PBMCs from patients with AR were co-cultured with allogeneic mDCs, sEV-mDCs or sEV-mDCs with MF63 (1 μM) for 3 days, Intracellular IL-13 levels in ILC2s were analyzed by flow cytometry. B. The levels of IL-13 in the supernatants of co-cultures with or without anti-IL-10 antibody detected by ELISA. *P< 0.05.


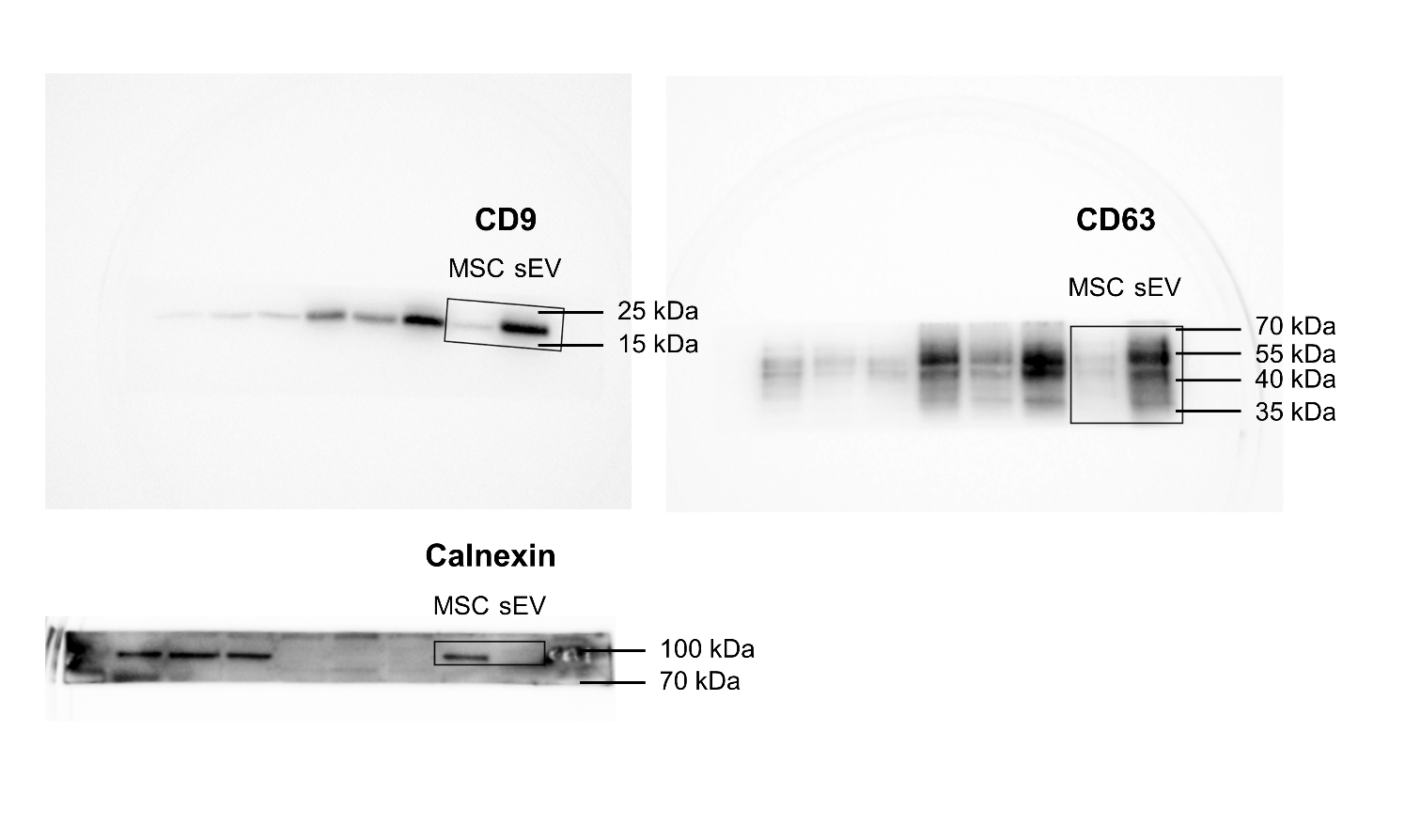


**Supplementary Fig. 4 Original uncropped blots for Fig. 1C.**
